# Cavitation dose painting for focused ultrasound-induced blood-brain barrier disruption

**DOI:** 10.1038/s41598-019-39090-9

**Published:** 2019-02-26

**Authors:** Yaoheng Yang, Xiaohui Zhang, Dezhuang Ye, Richard Laforest, Jeffrey Williamson, Yongjian Liu, Hong Chen

**Affiliations:** 1 Department of Biomedical Engineering, Washington University in St. Louis, Saint Louis, MO 63130 USA; 2 Mallinckrodt Institute of Radiology, Washington University School of Medicine, Saint Louis, MO 63110 USA; 3 Department of Mechanical Engineering and Materials Science, Washington University in St. Louis, Saint Louis, MO 63130 USA; 4 Department of Radiation Oncology, Washington University School of Medicine, Saint Louis, MO 63108 USA

## Abstract

Focused ultrasound combined with microbubble for blood-brain barrier disruption (FUS-BBBD) is a promising technique for noninvasive and localized brain drug delivery. This study demonstrates that passive cavitation imaging (PCI) is capable of predicting the location and concentration of nanoclusters delivered by FUS-BBBD. During FUS-BBBD treatment of mice, the acoustic emissions from FUS-activated microbubbles were passively detected by an ultrasound imaging system and processed offline using a frequency-domain PCI algorithm. After the FUS treatment, radiolabeled gold nanoclusters, ^64^Cu-AuNCs, were intravenously injected into the mice and imaged by positron emission tomography/computed tomography (PET/CT). The centers of the stable cavitation dose (SCD) maps obtained by PCI and the corresponding centers of the ^64^Cu-AuNCs concentration maps obtained by PET coincided within 0.3 ± 0.4 mm and 1.6 ± 1.1 mm in the transverse and axial directions of the FUS beam, respectively. The SCD maps were found to be linearly correlated with the ^64^Cu-AuNCs concentration maps on a pixel-by-pixel level. These findings suggest that SCD maps can spatially “paint” the delivered nanocluster concentration, a technique that we named as cavitation dose painting. This PCI-based cavitation dose painting technique in combination with FUS-BBBD opens new horizons in spatially targeted and modulated brain drug delivery.

## Introduction

Focused ultrasound in combination with microbubbles for blood-brain barrier disruption (FUS-BBBD) has been established as a promising technique for the delivery of therapeutic agents to a targeted brain location without invasive surgery^1–3^. The blood-brain barrier (BBB) excludes ~100% of large-molecule drugs and 98% of small-molecule drugs from entering the brain parenchyma^4^. FUS-BBBD utilizes the mechanical interactions between microbubbles and the surrounding blood vessels to enhance the BBB permeability, allowing various therapeutic agents in the systemic circulation to be delivered across the BBB to the brain tissue. Extensive preclinical studies have been performed to develop the FUS-BBBD technique, including evaluation of its feasibility and safety in smaller animal models (*e*.*g*., mice, rats, and rabbits)^3,5–10^ and larger animal models (*e*.*g*., pigs and non-human primates)^11,12^; optimization of treatment protocols^13^; assessment of therapeutic efficacy for various diseases^5,6,9^; and revealing its fundamental mechanisms^14–16^. Recently, a clinical trial successfully demonstrated the feasibility and safety of FUS-BBBD in patients with Alzheimer’s disease^17^. Despite these great advancements of the FUS-BBBD technique, there is an unmet need for a treatment monitoring technique that is capable of predicting the location and concentration of therapeutic agents delivered by FUS-BBBD. This capability is critically needed to verify localized drug delivery to the targeted brain location without off-target effects in the brain; assess whether the concentrations of the delivered agents reach the desired therapeutic levels; characterize variations among repeated treatments; and develop real-time feedback control of the FUS parameters to deliver the desired concentrations of drugs to the targeted locations.

Several existing imaging techniques have been used for the assessment of FUS-BBBD treatment outcome. Magnetic resonance imaging (MRI) is the most commonly used imaging modality for the assessment of FUS-mediated BBB permeability changes based on the leakage of MR contrast agents from the blood into the brain parenchyma. Positron emission tomography (PET) and single photon emission computed tomography (SPECT) in combination with radiolabeled therapeutic agents provide noninvasive, sensitive, and quantitative methods for directly assessing the spatial distribution of radiolabeled agents^18–20^. Several studies have used PET or SPECT to evaluate the spatial distributions of radiolabeled agents in the brain after FUS-BBBD treatment^21–23^. Our previous study verified that *in vivo* PET imaging can reliably estimate the concentrations of the ^64^Cu-labeled gold nanoclusters (^64^Cu-AuNCs) delivered to the brain using *ex vivo* gamma counting and inductively coupled plasma mass spectrometry (ICP-MS)^20^. The ^64^Cu radioactivity was found to be linearly correlated with the Au concentration quantified using ICP-MS (*R*^2^ = 0.94), which confirmed the radiolabel stability of ^64^Cu for accurate measurement of ^64^Cu-AuNCs distribution^20^. However, MRI, PET, and SPECT cannot be used to perform intraprocedural monitoring of the FUS treatment.

Among existing techniques, passive cavitation monitoring, including passive cavitation detection (PCD) and passive cavitation imaging (PCI), is the most commonly used technology for FUS-BBBD treatment monitoring. The underlying concept is that mechanical oscillations of the microbubbles emit ultrasound waves that can be detected using ultrasound sensors to passively “listen” to the secondary acoustic emissions by the microbubbles^24^ (this differs from active pulse-echo ultrasound imaging). Passive cavitation detection (PCD), using a single-element ultrasound sensor, has been broadly used for real-time FUS-BBBD treatment monitoring to quantify cavitation levels, differentiate between cavitation modes (*i*.*e*., stable cavitation and inertial cavitation), investigate bioeffects of cavitation, and develop feedback control algorithms to enhance the FUS treatment safety and efficacy^25–27^. Stable cavitation (SC, small amplitude microbubble oscillation) can be characterized by the detection of harmonic emissions in the frequency domain, while inertial cavitation (IC, large amplitude microbubble oscillation followed by violent collapse) can be characterized by broadband emissions^28–30^. PCD has been implemented for real-time monitoring of the FUS-BBBD treatment^27,31,32^ and controlling FUS exposure parameters using a closed-loop control algorithm^27^. Although PCD has been shown to be a useful tool for FUS-BBBD monitoring, it cannot detect the spatial distribution of cavitation.

PCI, integrating multi-element ultrasound arrays with beam-forming techniques, can be used to image the spatial distribution of cavitation. Using available ultrasound imaging probes, different 2D PCI algorithms have been introduced, for example, time-domain delay-and-sum beamformer^33^, time-domain robust Capon beamformer^34^, frequency domain delay-and-sum beamformer^35^, robust beamforming by linear programming^36^, and the angular spectrum method^37^. Moreover, a recent study demonstrated the feasibility of 3D passive cavitation imaging using a customized 2D array^38^. However, to our knowledge, only two studies have been published on evaluating the feasibility of using PCI to predict the location of BBBD by correlating PCI with the BBB permeability changes evaluated by post-treatment contrast-enhanced MRI^39,40^. No study has been reported on using PCI to estimate the spatial distribution (both location and concentration) of therapeutic agents delivered by FUS-BBBD.

The objective of this study was to evaluate the potential of PCI for estimating the location and concentration of therapeutic agents delivered by FUS-BBBD. ^64^Cu-AuNCs were used as model agents because they are chemically stable, biocompatible, easily functionalized by labeling, and constitute a promising theranostic nanomedicine in their own right^41–43^. *In vivo* PET imaging was used to quantify the spatial distribution of ^64^Cu-AuNCs in the brain after FUS-BBBD treatment and correlate it with the cavitation activity detected by PCI during FUS-BBBD treatment. We demonstrated that PCI was capable of pixel-wise estimation of the delivered ^64^Cu-AuNCs concentration, a technique that we named as cavitation dose painting.

## Results

**Figure 1:**
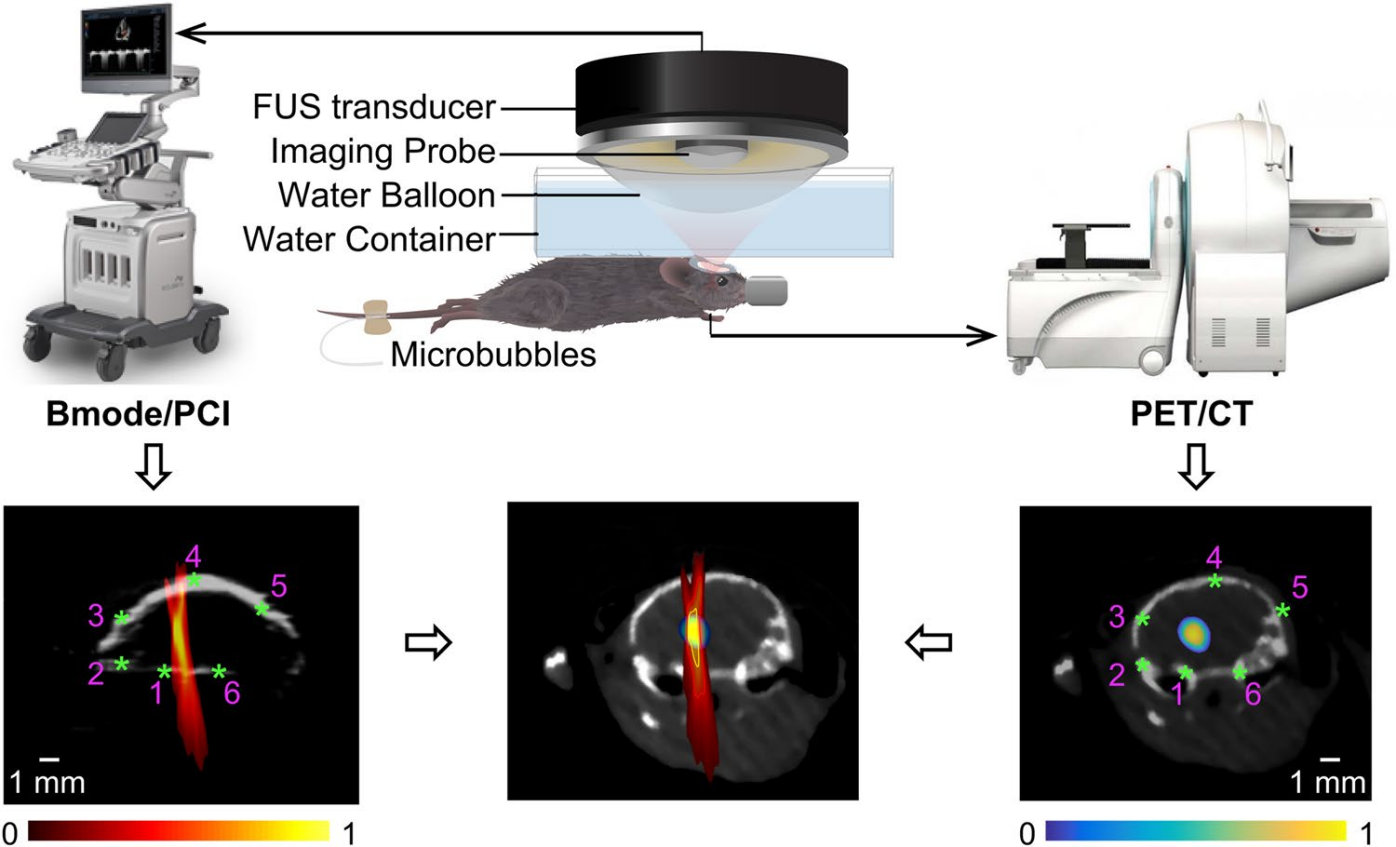
Illustration of the FUS system, as well as images acquisition and registration methods. PCI was acquired during FUS treatment, and PET/CT image was acquired 24 h after FUS treatment. The registration between PCI and PET was performed based on the shared anatomic feature of the skull (indicated by the asterisks) in both B-mode and CT images.

**Figure 2:**
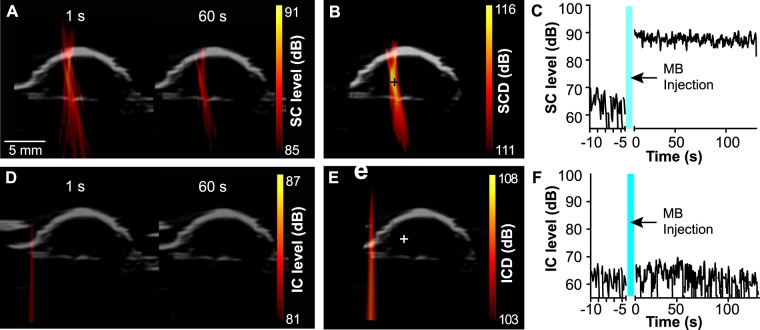
Representative SC and IC maps. (**A)** SC level maps obtained during FUS treatment at two different time points (1 s and 60 s). **(B)** SCD map acquired by integrating the SC level for reach pixel through the total FUS treatment time. **(C)** Representative SC level-time curve for the pixel identified by the cross in B. **(D–F)** Corresponding IC level maps, ICD map, and IC level-time curve.

Using an experimental setup illustrated in Fig. 1, PCI successfully recorded the spatiotemporal dynamics of microbubble cavitation activity in the mouse brain during FUS treatment. Figure 2A displays representative SC level maps (used to quantify the stable cavitation activity at individual time points) acquired at different time points (1 s and 60 s) during the treatment. PCI recorded the spatial distribution of the cavitation activity over time. Figure 2B shows the corresponding SCD map, which was generated by integrating the SC level over the whole FUS treatment duration for each pixel to quantify the total cavitation energy. Figure 2C shows the SC level over time at one representative pixel (indicated by the cross in Fig. 2B). The SC level increased immediately after bolus injection of the microbubbles and then showed the trend of decrease over time. Figure 2D–F present the corresponding IC level map, IC dose (ICD) map, and IC level-time curve, which all showed that the microbubble IC activity was at the noise level. PCI data was acquired successfully in eight out of nine mice. The failure to perform PCI in one mouse was due to the malfunction of the ultrasound imaging system.

**Figure 3:**
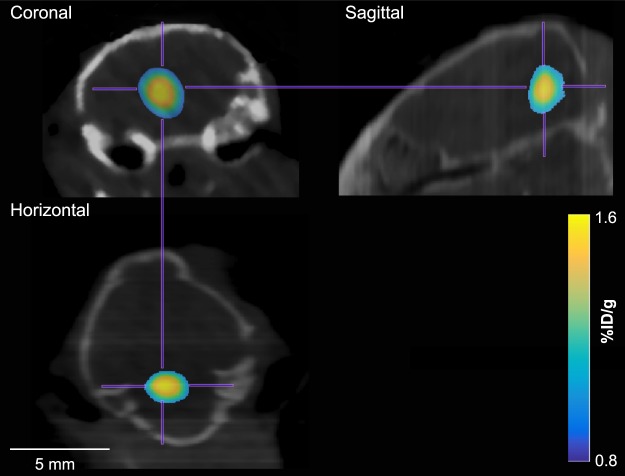
Representative PET images of a mouse in the coronal, sagittal, and horizontal planes.

Same as our previous publications^19,20^, PET was used to quantify the concentrations of ^64^Cu-AuNCs delivered by FUS-BBBD in terms of the percent injected dose per gram of tissue (%ID/g). Successful delivery of ^64^Cu-AuNCs at the FUS-treated brain region was observed in seven out of the eight mice with successful PCI data acquisition, as indicated by higher radioactivity at the targeted brain location compared with surrounding untreated brain location (Fig. 3). The failure to deliver ^64^Cu-AuNCs to one mouse was due to an unsuccessful tail vein injection indicated by the accumulation of radioactivity in the mouse tail on the acquired PET image. Results obtained from this mouse was discarded in the analysis. Therefore, we have a total of seven mice for analyzing the correlation between PCI and PET.

**Figure 4:**
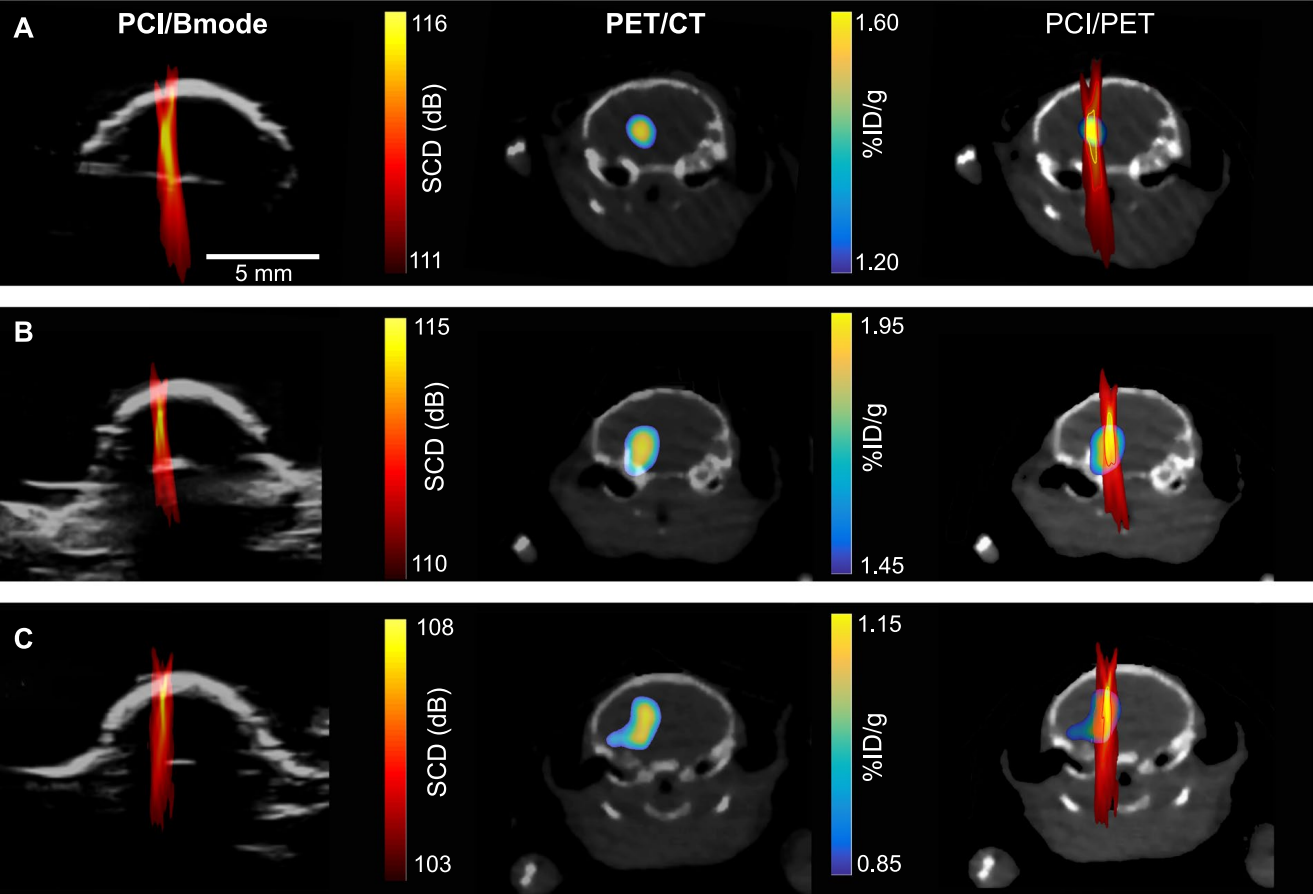
PCI/B-mode images (left column), PET/CT images (middle column), and PCI and PET overlaid images (right column) for three representative cases shown in **A**, **B**, and **C**, respectively.

Figure 4 displays representative SCD maps (column 1), ^64^Cu-AuNCs concentration maps acquired at approximately the same imaging plane as the SCD maps (column 2), and overlay of these two maps (column 3) for three representative cases. Although all seven mice showed localized delivery of the ^64^Cu-AuNCs in the mouse brains after FUS-BBBD treatment using the same protocol, variations in the recorded microbubble cavitation activity and the delivery outcomes were observed among mice as shown by these representative cases. For the three cases, the maximum SCD of each case varied from 108 dB to 116 dB, and the maximum ^64^Cu-AuNCs concentration of each case varied within the range of 1.15–1.95%ID/g.

**Figure 5:**
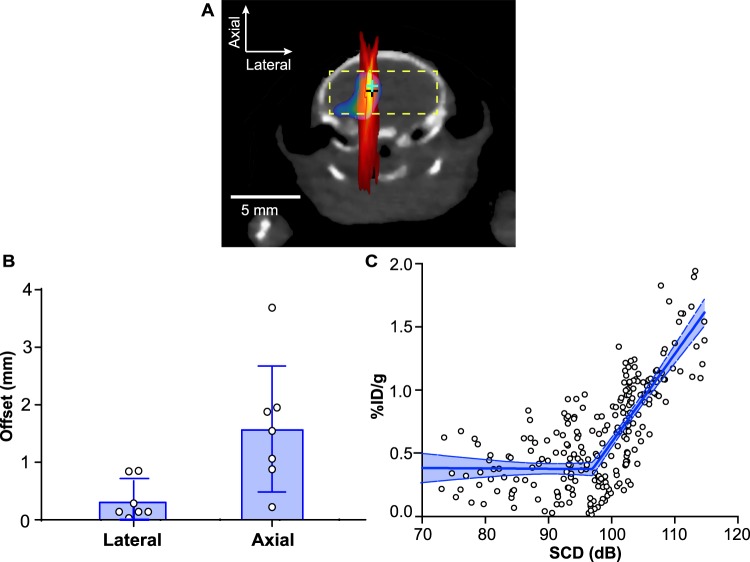
Quantitative analysis of the spatial and dose correlation between SCD maps and ^64^Cu-AuNC concentration maps. **(B)** The offsets between the pixel with maximum SCD and the pixel with the maximum ^64^Cu-AuNC concentration (indicated by crosses in **A**). (**C)** A pixel-by-pixel correlation between SCD maps and ^64^Cu-AuNC concentration maps within the 2D region in the brain (indicated by the rectangular box in **A**). Error bar in **(B)** represents standard deviation. Shaded blue region in **(C)** represents the 95% confidence level.

For all the treated mice, the offset between the pixel with the maximum SCD (SCD_max_) on the PCI maps and the pixel with the highest concentration of ^64^Cu-AuNCs on the PET images were 0.3 ± 0.4 mm and 1.6 ± 1.1 mm in the transverse and axial directions of the FUS beam, respectively (Fig. 5B). We then evaluated the pixel-by-pixel correlation between the SCD maps and the concentration maps within a region in the brain (the width of this region was defined by a lateral line within the brain passing the SCD_max_; the height of this region was defined by −1 dB of the SCD_max_ in the axial direction to ensure this region was inside the brain). A good linear correlation was obtained based on segmented linear regression (*R*^2^ = 0.61). The segmented linear regression was used to model a threshold for BBBD in reference to a previous publication^27^.

**Figure 6:**
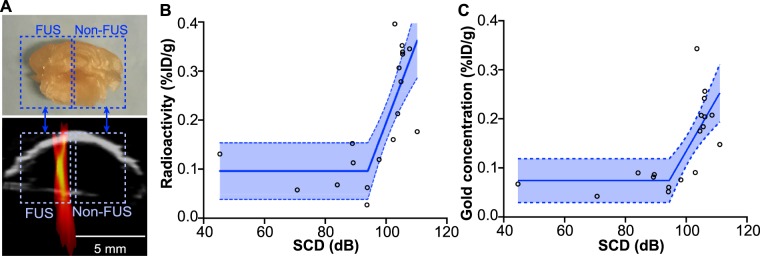
(**A)** The *ex vivo* mouse brains were sliced coronally into 2-mm slices, and the slice containing the targeted brainstem was cut into two halves for quantifying the radioactivity in each half (illustrated by the rectangle boxes). The SCD was also averaged within each half of the brain for identifying the correlation between SCD and radioactivity. (**B**) The radioactivity of ^64^Cu-AuNCs quantified using gamma counting and **(C)** the gold concentration of ^64^Cu-AuNCs quantified using ICP-MS in the FUS-treated halves and contralateral non-treated halves were well correlated with the corresponding spatial-averaged SCD within the same brain regions using the segmented linear regression (*R*^2^ = 0.62 for gamma counting and *R*^2^ = 0.53 for ICP-MS).

To validate the above correlation between PCI and PET identified *in vivo*, nine additional mice were treated by the same protocol and sacrificed at 24 h after FUS treatment for the *ex vivo* quantification of the radioactivity using gamma counting and measurement of gold concentration using ICP-MS (Fig. 6A). It was found that the ^64^Cu-AuNCs concentration quantified *ex vivo* in the FUS-treated halves and contralateral non-treated halves were well correlated (*R*^2^ = 0.62 for gamma counting and *R*^2^ = 0.53 for ICP-MS) with the spatial-averaged SCD within the corresponding brain regions using the segmented linear regression (Fig. 6B,C).

## Discussion

This study demonstrated that PCI was capable of predicting the location and concentration of ^64^Cu-AuNCs delivered by FUS-BBBD, a technique that we named as cavitation dose painting. This PCI-based cavitation dose painting technique can be used in the future to guide FUS-BBBD treatment to achieve conformal delivery of therapeutic agents to the targeted brain region and avoid side effects in the non-targeted brain area, and to deliver higher prescribed drug concentrations to high-risk subvolumes while treating lower-risk subvolumes by delivering lower prescribed drug concentrations. This novel strategy can open new horizons in controlled and precise brain drug delivery.

The capability of PCI to measure the location of FUS-BBBD mediated drug delivery was evaluated by quantifying the offsets between the pixel location of the SCD_max_ and the pixel location with the highest concentration of ^64^Cu-AuNCs. The spatial resolution of PCI is determined by the diffraction pattern of the imaging array. The lateral resolution of PCI is at the scale of 10x of the axial resolution, which limits the accuracy of PCI in predicting the agent delivery location in the axial direction. Two previous studies evaluated the capability of PCI in estimating the location of the BBB disruption by comparing PCI with post-treatment contrast-enhanced MRI^39,40^. In one study, the mean offsets were 0.7 mm and 2.2 mm in the transverse and axial directions, respectively^40^. In the other study, it was reported that the offsets were 0.3 ± 1.5 mm and 0.5 ± 7.5 mm in the transverse and axial directions, respectively^39^. Although the offsets were close to those found in the current study (0.3 ± 0.4 mm in the transverse direction and 1.6 ± 1.1 mm in the axial direction), the two previous studies used contrast-enhanced MRI to infer the locations of the delivered agents. The physicochemical properties of MR contrast agents are different from the actual agents intended to be delivered by FUS-BBBD. Thus, the biodistribution of MR contrast agents may not accurately represent the distribution of the delivered agent. The present study directly imaged the ^64^Cu-AuNCs distribution by PET. PET imaging of radiolabeled nanoparticles provides a noninvasive, highly sensitive, and quantitative method for assessing the efficiency of nanoparticle delivery and their spatial distribution. Therapeutic agents beside nanoparticles, *e*.*g*., chemotherapy drugs, peptides, and proteins, can be radiolabeled for directly evaluating the agent spatial distribution in the brain using PET^44^, instead of inferring its spatial distribution based on the leakage of MR contrast agents.

Our study showed for the first time that a linear relationship exists between SCD quantified using PCI (2D) and ^64^Cu-AuNC concentration quantified using PET at a pixel-by-pixel level. A previous study also found a similar linear correlation between SCD quantified using PCD (1D) and the concentration of the delivered agents quantified using *ex vivo* fluorescence imaging. As mentioned earlier, our previous study demonstrated that the ^64^Cu radioactivity was linearly correlated with the Au concentration (*R*^2^ = 0.94), indicating that the radioactivity of ^64^Cu can accurately measure the concentration of the AuNCs^20^. In the current study, the linear relationship identified using *in vivo* PET was verified with *ex vivo* quantification of the radioactivity using gamma counting (Fig. 6B) and *ex vivo* quantification of the gold concentration using ICP-MS (Fig. 6C). As the *ex vivo* study measured the average ^64^Cu-AuNC concentration in each half of the brain slices (Fig. 6A), the values of the measured concentrations (Fig. 6B,C) were lower than those obtained using PET (Fig. 5C).

All the mice were treated using the same FUS parameters; however, the cavitation dose maps and ^64^Cu-AuNCs delivery outcomes varied (Fig. 4). This variation among repeated FUS-BBBD treatment has been reported before. It can be caused by variation among repeated experiment in parameters that are hard to control, such as the size distribution of microbubbles reaching the targeted brain location^45^, circulating microbubble concentration in blood^46^, blood vessel density within the treated region of the brain^47^, and heterogeneous acoustic property of skull^48^. This finding justified the need for treatment monitoring to characterize variation among experiments.

Our finding that PCI can quantitatively measure the location and concentration of ^64^Cu-AuNCs delivered by FUS-BBBD is significant because it suggests that PCI can be used to verify localized drug delivery to the targeted brain site without off-target effects in the brain, assess whether the concentration of the delivered agent reaches the desired therapeutic level, and characterize variations among repeated treatments. Real-time PCI needs to be developed in the future to quantify the cavitation dose during the FUS-BBBD treatment and provide feedback control of the FUS parameters during the treatment. This PCI-feedback-controlled FUS-BBBD technique will allow controlling drug delivery location and spatially tailoring the delivered drug concentration to match the needed drug delivery doses at different subvolumes of the diseased brain region. The cavitation dose painting technique is not limited to the delivery of nanoparticles. In future clinical translation, therapeutic agents (*e*.*g*., chemotherapeutic drugs, peptides, and proteins) can be radiolabeled and injected to the patient at a low dose (lower than the therapeutic level) after FUS-BBBD treatment. The relationship between SCD and the delivered drug concentration as quantified by PET can be identified to develop the cavitation dose painting technique, which can then be used to estimate and control the delivery of the actual drugs without radiolabeling.

This proof-of-concept study demonstrated the concept of cavitation dose painting. Future work is needed to address several limitations of this study. First, PCI imaged cavitation activity in one coronal plane while PET imaged the radioactivity of the delivered ^64^Cu-AuNCs in 3D. Future studies are needed to improve the spatial co-registration of PCI and PET by developing 3D PCI techniques. Second, the pixel size of the PET images was 0.8×0.8 mm^2^ and the pixel size of the PCI was 0.2 × 0.2 mm^2^. Downsampling of the PCI was performed in order to analyze the pixel-by-pixel correlation between PCI and PET. Future studies using larger animal models (*e*.*g*., pigs and non-human primates) will allow us to better characterize the pixel-wise correlation between these two imaging modalities. Third, although almost the whole brain was included in our pixel-by-pixel analysis (Fig. 5C), we did not use the whole brain region as our region of interest because the axial resolution of PCI was low and including regions outside the −1 dB of the SCD_max_ in the axial direction decreased the correlation coefficient to *R*^2^ = 0.45. Future studies need to improve the axial resolution of PCI by developing advanced PCI imaging algorithms^36^.

## Materials and Methods

### FUS-BBBD treatment

All animal procedures were reviewed and approved by the Institutional Animal Care and Use Committee of Washington University in St. Louis, in accordance with the National Institutes of Health Guidelines for animal research. C57BL/6 female mice (6–8 weeks, 20–25 g body weight, n = 18 in total) were purchased from Charles River Laboratory (Wilmington, MA, USA). Following a procedure described in our previous publication^20^, the animals were prepared for FUS sonication by removing the fur on the mouse head and coupled to a water container using ultrasound gel.

The prepared mice were sonicated using a commercially available ultrasound image-guided FUS system (VIFU 2000, Alpinion, Bothell, WA, USA) that integrated a FUS system with a software programmable ultrasound imaging system. This system used a FUS transducer with a center frequency of 1.5 MHz, a focal depth of 60 mm, an aperture of 60 mm, and a circular central opening of 38 mm. A linear array (L8–17, Alpinion, Seoul, Korea) with a bandwidth of 8–17 MHz and a center frequency of 12 MHz was inserted into the FUS transducer center opening. The ultrasound imaging plane was aligned with the FUS focal plane.

The ultrasound imaging system was used for both treatment planning and PCI. Treatment planning was performed with the assistant of a metal grid using a previously published method to align the FUS transducer focus at the left brainstem^20^. Size-isolated microbubbles with a median diameter of 4–5 μm were prepared in-house according to a previously described protocol^45^ and diluted using sterile saline to a final concentration of about 8 × 10^8^ number of microbubbles per ml. The diluted microbubbles (volume = 25 μL) were administered by a bolus injection *via* the tail vein. Immediately after injection (~9 s), the mice were treated by the FUS system using the following parameters: center frequency = 1.5 MHz, peak negative pressure = 0.61 MPa, pulse length = 6.7 ms, pulse repetition frequency = 5 Hz, and sonication duration = 120 s. After treatment, mice were put back into their cages for them to recover from anesthesia.

The pressure amplitude and beam dimensions of the FUS transducer were calibrated using a needle hydrophone (HGL-0200, Onda, CA, USA) in a degassed water tank before the experiment. The pressures reported here were the measured hydrophone peak negative pressures corrected for 18% mouse skull attenuation^3^. The full width at half maximum (FWHM) dimensions of the axial and lateral beams were 6.04 mm and 0.62 mm, respectively.

### PCI data acquisition and processing

The ultrasound imaging system was programmed to first operate in the pulse-echo mode to acquire a B-mode image for identifying the location of the mouse skull during PCI post-processing. The system was then operated at the passive mode for acquiring two sets of PCI data. The first PCI data acquisition was performed with FUS sonication on but before the injection of microbubbles to define the background cavitation noise level. The second PCI data acquisition was performed immediately after microbubble injection and in synchronized with the FUS sonication. During the sonication of each FUS pulse, one PCI frame was acquired with an acquisition duration of 400 μs.

In reference to a PCI method used by Haworth *et al*.^35^, a frequency-domain PCI algorithm was written in Matlab (Mathworks inc., Natick, MA, USA) to proccess the acquired PCI data offline through the following procedure: (1) Apply Butterworth bandpass filtering around the imaging probe bandwidth of 8–17 MHz to reduce contributions from FUS source signals; (2) Apply a phase shift to the signal acquired by each element in the frequency domain (equivalent to a time delay in temporal domain) based on the propagation times between the receiving element and the spatial location the pixel represents; (3) Sum the phase-shifted waveforms across all the elements for each pixel and its energy is computed by calculating the square of the summed waveforms; Parallel computation using Graphics Processing Units (GPUs) was integrated into the Matlab code to accelerate the calculation speed; (4) Calculate the mean amplitude of the spectrum within selected harmonic bandwidths (0.3 MHz window around all harmonics and superharmonics) for each pixel to generate SC maps; the mean amplitudes of braodband signal falling within frequency bands between the harmonic and ultraharmonic bands were used to calculate the IC levels and generate the IC maps; (5) The SC and IC maps obtained from each FUS treatment were integrated over time to obtain the SCD and ICD maps, respectively.

### *In vivo* MicroPET/CT image acquisition and processing

The mice were imaged by the Inveon PET/CT system (Siemens, Knoxville, TN) at 24 h after intravenous injection of ^64^Cu-AuNCs at a concentration ~10 MBq following the FUS-treatment. Details on the ^64^Cu-AuNCs manufacture and characterization were reported in our previous publications^49^. The ^64^Cu-AuNCs had a homogeneous size distribution with the hydrodynamic diameter = 5.60 ± 0.50 nm and zeta potential = −0.40 ± 0.11 mV. In our previous study, we performed PET imaging at 1 h, 4 h, and 24 h post treatment and found the ^64^Cu-AuNCs were almost cleared from the blood circulation at the 24 h time point^20^. Therefore, in the current study, we chose to perform the microPET/CT scans at the 24 h time point as it allowed us to more accurately quantify the ^64^Cu-AuNCs concentrations in the brain tissue compared with earlier time points by eliminating the interference of the ^64^Cu-AuNCs in the blood.

The acquired PET images were corrected for attenuation, scatter, normalization, and camera dead time and co-registered with CT images. The PET images were reconstructed with the maximum a posteriori (MAP) algorithm. Images were analyzed using Inveon Research Workplace (Siemens, Knoxville, TN) and the Matlab program. Partial volume correction was performed following an established method to eliminate the spillover of signals from tissue outside the brain^50^. Decay correlation was applied to compensate for the decay of ^64^Cu over time. The pixel intensity of the PET image was quantified as percent injected dose per gram tissue (%ID/g).

### Analysis of the correlation between PCI and PET

Since the ultrasound imaging probe was co-axially aligned with the FUS transducer, the focal point of the FUS transducer was located in the ultrasound imaging plane. To investigate the correlation between PCI and PET, we first identified the PET voxel location with the highest radioactivity inside the brain, which was considered to be the location of the FUS focus. Then the PET images acquired in the coronal plane passing through this voxel was selected and considered to be the corresponding imaging plane for the B-mode/PCI. This selection of the corresponding PET imaging plane was verified in all the cases as the same anatomic features of the mouse skulls were observed in the B-mode images and corresponding CT images (Fig. 1). Then six controlling points were selected on the B-mode and corresponding CT images (Fig. 1). The CT images were translated and rotated to fit the same controlling points on B-mode images. Through this process, the transformation matrix was acquired and applied to PET images to register the PET images with PCI images.

For each mouse, the capability of SCD in predicting ^64^Cu-AuNC delivery location was characterized by quantifying the offsets between the pixel location of the SCD_max_ and the location with the maximum ^64^Cu-AuNC concentration. The offsets of these two locations were decomposed to the lateral direction and axial direction. The capability of SCD in predicting ^64^Cu-AuNCs concentration was determined by evaluating the pixel-by-pixel correlation between the SCD map and the concentration map. The pixel size of the PET scanner was 0.8 mm × 0.8 mm, corresponding to the intrinsic sampling size of the scanner. The SCD maps had a pixel size of 0.2 mm × 0.2 mm. To perform a pixel by pixel comparison between these two maps, we first aligned the two images by aligning the pixel location of SCD_max_ and the pixel with the highest concentration. Then we decreased the pixel size of the SCD map by downsampling to match that of the PET. The correlations between these two maps within the 2D region (Fig. 5A) were evaluated by the segmented linear regression using GraphPad Prism (Version 6.04, La Jolla, CA, USA), and the goodness of fit was assessed by the correlation coefficient, *R*^2^.

### *Ex vivo* quantification

To validate the correlation found between PCI and *in vivo* PET imaging, nine additional mice were used to perform *ex vivo* quantification of ^64^Cu-AuNCs radioactivity using gamma counting and Au concentration by ICP-MS^20^. These mice were treated using the same FUS treatment protocol as described in 4.1. After FUS treatment, they were sacrificed by transcardial perfusion at 24 h after FUS treatment; their brains were collected and sliced coronally into 2-mm slices. Then the slices containing the brainstem was cut into two halves (left and right representing FUS-treated and non-treated samples, respectively) and the radioactivity was counted using a Beckman 8000 gamma counter (Beckman, Fullerton, CA). The count rate (counts per minute, CPM) for each tissue sample was corrected by automatic background subtraction. Decay correction was applied (compensated for the decay of ^64^Cu radioactivity over time). The corrected CPM from each tissue sample was normalized both to the mass of the tissue sample (in grams, g) and to the injected dose (ID). The concentration of ^64^Cu-AuNCs in each tissue sample was then calculated as %ID/g. The correlation between the radioactivity (%ID/g) of ^64^Cu-AuNCs in the FUS-treated and contralateral non-treated brainstem and the corresponding spatial-averaged SCD within the same regions was evaluated using the segmented linear regression.

After the gamma counting, these samples were then digested using a high-pressure microwave digestion system (Milestone Inc. Monroe, CT) and the gold concentrations in the digested brain tissue samples were determined using ICP-MS (Elan DRC-e, PerkinElmer, Germany). Au standard was used to generate the standard curve. The Au concentration was expressed as the percentage of the Au in the brainstem over the total injected Au normalized by the tissue weight (%ID/g). The Au concentration (%ID/g) in FUS-treated and non-treated brainstem was correlated with the corresponding spatial-averaged SCD within the same regions using the segmented linear regression.

## Conclusions

This paper has demonstrated the potential of PCI in predicting the spatial location and concentration of therapeutic agents delivered by FUS-BBBD. We call this new strategy PCI-based cavitation dose painting. The accuracy of this strategy was evaluated by correlating PCI with *in vivo* PET/CT imaging of radiolabeled nanoparticles delivered by FUS-BBBD. This study laid the foundation for the future development of PCI-based real-time feedback control of the FUS-BBBD treatment to spatially control and modulate the delivered drug concentrations.

## Data Availability

The datasets generated in this study are available from the corresponding author upon request.
